# Effects of conventional versus 3D-printed cosmetic covers on user satisfaction and psychosocial well-being in lower limb prostheses users: A randomised crossover trial

**DOI:** 10.1177/20556683251330996

**Published:** 2025-04-04

**Authors:** Nerrolyn Ramstrand, Maria Riveiro, Lars Eriksson, Michael Ceder

**Affiliations:** 1 Department of Rehabilitation, Jönköping University, Jönköping, Sweden; 2 Department of Computer Science and Informatics, Jönköping University, Jönköping, Sweden; 3 Department of Product Development, Production and Design, Jönköping University, Jönköping, Sweden

**Keywords:** amputation, prosthetic limb, cosmesis, design, outcome measurement

## Abstract

**Introduction:**

The objective of this study was to evaluate the effects of prescribing a traditional foam cosmetic cover versus a more recently developed 3D printed cosmetic cover on the satisfaction and psychosocial wellbeing of prosthesis users.

**Methods:**

Transtibial and transfemoral prosthesis users were randomly assigned into two groups. One group was fitted with a foam cosmesis with a nylon stocking while the other received a 3D printed cosmetic cover. Cosmeses were worn for 12 weeks before being switched to the alternate design. Outcomes related to satisfaction and psychosocial wellbeing (ABIS-R, TAPES, QUEST) were collected on 3 occasions. Linear mixed effects models assessed for differences between the cosmetic covers.

**Results:**

10 participants completed all outcome measures on 3 occasions. Significant differences in favour of the 3D printed cosmesis were observed for TAPES general psychosocial adjustment (*p* = .03), TAPES aesthetic satisfaction (*p* = .04) and ABIS-R (*p* = .025). Adjustment to physical limitations were higher for the foam cover (*p* = .008). No differences were observed in QUEST scores. Covariates; age, time since amputation, extroversion, did not have any significant effects.

**Conclusion:**

Results suggest that cosmetic cover design can significantly affect prosthesis users’ psychosocial wellbeing and satisfaction with aesthetic appearance. Variance between participants is high indicating diverse preferences.

## Introduction

The impact of a major limb amputation on an individual’s psychosocial wellbeing is a major healthcare issue that can be positively influenced by the prescription of a prosthetic device.^1,2^ Prosthetic related factors that have been linked to the psychosocial wellbeing of people with limb amputations include the comfort of the device,^1^ walking capacity,^1^ hours of use,^1,3^ satisfaction with weight of the device^4^ and satisfaction with aesthetic appearance.^5–7^ Over the past decade there has been growing interest in the aesthetic design of prostheses^7–9^ and a substantial increase in the options available to prosthesis users.^10^ Despite significant advancements and an increasing number of cosmetic options, people who have undergone amputations continue to express dissatisfaction with the appearance of their prostheses.^1,6,7^

Satisfaction with the aesthetic appearance of a prosthetic limb is an important factor associated with psychosocial adjustment following an amputation^1,3^ and has been demonstrated to affect perceptions of body image,^11,12^ self-acceptance^13^ and prosthesis embodiment.^5^ There are also indications that satisfaction with aesthetics impacts upon prosthesis users’ level of participation in social situations.^2,5,14,15^ Satisfaction has been reported as poorer in females as compared to males,^6^ while younger prosthesis users report lower levels of satisfaction than older users.^6^ Little is known of how specific aesthetic design characteristics influence clients perceptions of the device however, it has been suggested that prostheses with a more realistic appearance are associated with higher levels of satisfaction.^6^ This is supported by the finding of Donovan-Hall et al., who demonstrated that 12 weeks of using a silicon cosmetic cover significantly increased users’ level of engagement in activities that involved revealing their bodies.^13^ Dissatisfaction with the colour, touch/feel, shape and weight of the device have been highlighted as design properties associated with lower levels of satisfaction.^6,16^

The aesthetic appearance of prostheses is manipulated using different designs of cosmetic cover. Vlachaki et al.^17^ have classified aesthetic designs into three distinct categories, 1/Realistic prostheses which imitate the natural appearance of a human limb, 2/functional prostheses in which base components are exposed and no cosmetic cover is used and 3/expressive prostheses which incorporate different colours, textures and finishes into a cosmetic cover. Hall et al.^18^ propose that preference for a specific aesthetic design is related to the way in which an individual wishes to communicate their sense of self, how they view themselves and how they want to present themselves to others. In Sweden, the most commonly prescribed cosmetic cover for lower limb prosthesis users is a realistic design, manufactured from a foam block, shaped to mimic the form of an anatomical leg and covered with a nylon stocking or pull up skin. These ‘foam’ covers are considered affordable, light weight and allow for adjustment of the alignment without affecting the integrity of the prosthesis. They are however limited in terms of durability and customisation.^8^ With advancements in additive technology options for more expressive designs of cosmetic covers have increased substantially over the past decade and many companies now offer individually customisable 3D printed covers. Manufacture of these covers typically involves capturing the shape of a user’s prosthesis and sound leg using a 3D scan or series of photos and then allowing users to select their preference of colours and patterns.^19,20^ 3D printed cosmetic covers are generally more expensive than foam covers but considered beneficial in terms of ease of attachment, ease of cleanliness and reliability.^8^

To date the relative effects of different aesthetic designs on clients’ satisfaction with their device and their psychosocial wellbeing are poorly understood. The objective of this study was to evaluate the effects of two types of cosmetic covers, representing different design categories,^17^ on levels of satisfaction and psychosocial wellbeing in lower limb prosthesis users. The two covers chosen for evaluation were 1/a foam cover with nylon stocking, selected as it is the most common cover provided to prosthesis users in Sweden and because it represents the realistic category of covers and 2/a 3D printed cover, selected as it was considered to represent the newest alternative of covers on the market and because it represents the expressive category of covers.

## Methods

A randomised controlled crossover trial, using a two-period, two-sequence design (AB/BA) was used to evaluate prosthesis user satisfaction and psychological wellbeing when fitted with two different types of cosmetic cover. This design was selected over a traditional randomised parallel group design as it eliminates part of the inter-subject variability from the comparison and, by having participants serve as their own control, reduces the effect of covariates.^21^ The crossover design was also considered beneficial due to its high power, making it possible to achieve the same level of accuracy as a parallel design with fewer participants.^22^

**Figure 1.**
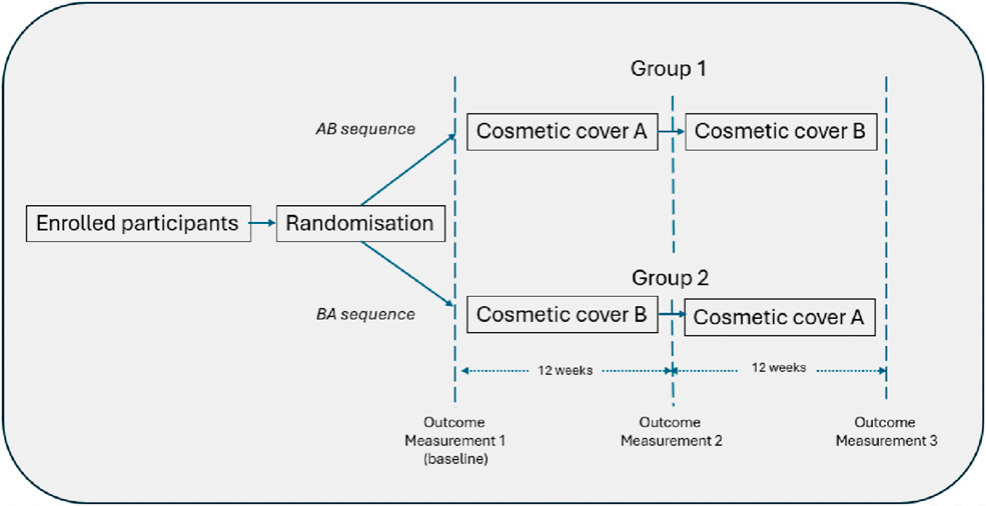
Two-period, two-sequence crossover design.

**Figure 2.**
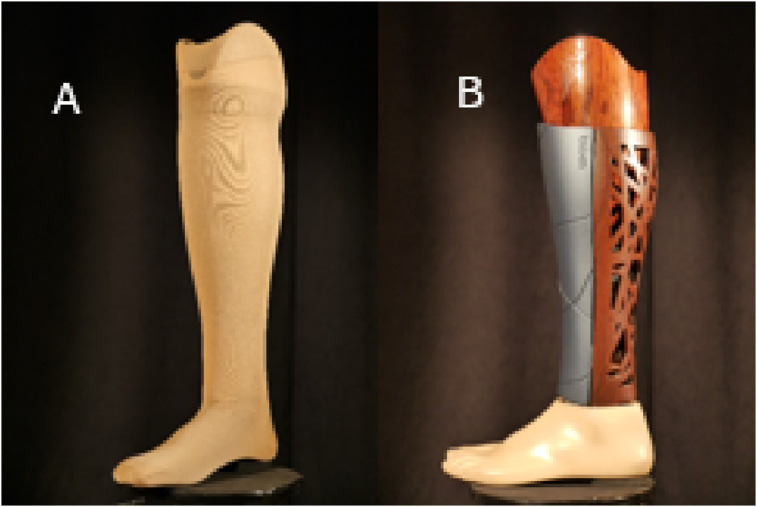
(a) Example of a traditional cosmetic cover, (b) Example of a 3D printed cosmetic cover.

The design of the study is presented as Figure 1. After recruitment and providing informed consent, participants were randomly assigned into two groups using a single sequence of random assignment (computer-generated random numbers). One group was initially fitted with a foam cosmetic cover formed from a plastazote material, or similar, and covered with a skin-coloured stocking (example Figure 2(a)). The second group was fitted with a 3D printed cosmetic cover (example Figure 2(b)). For the 3D printed cover participants were permitted to select a design of their choosing from the 3D flex, C-collection offered by UNYQ (UNYQ, Seville, Spain). After receiving the relevant cosmetic cover, participants were requested to wear their prosthesis for a period of 12 weeks. Their cover was then switched to the alternate design for a further 12-week period. No wash out period was included between testing occasions as carryover effects were anticipated as being minimal. Covers were provided at no cost to participants and they were informed at the beginning of the study that they would be permitted to keep their preferred cover upon conclusion of the study. Only cosmetic covers were altered during the study and other prosthetic componentry, e.g. foot, socket, remained unchanged.

### Participants

Participants were recruited from 9 different prosthetic and orthotic clinics in Sweden. They were recruited on the basis that they were over the age of 18 and had undergone a unilateral, trans-femoral, knee disarticulation or trans-tibial amputation at least 2 years prior to the study. To be eligible for participation they were also required to use a prosthesis daily and be able to ambulate outdoors, on stairs and on uneven surfaces. Prosthesis users who had previously expressed a desire to have a 3D printed cosmetic cover, or currently used a 3D cover, were not eligible to participate as this group of individuals would likely be biased from the outset.

All cosmetic covers were provided by the participants’ regular prosthetist. Each prosthetist received written instructions regarding the inclusion criteria and types of covers to provide. They also participated in a half-day training session. This training included information about the study, the importance of not showing a preference toward one type of cover over the other and information on how to scan/photograph participants when ordering a 3D cover. The study protocol was approved by the Swedish Ethical Review Authority (ref: 2023-01045-02).

### Outcome measures

All participants were requested to complete an outcome measures survey on three occasions;

(1) Outcome measurement 1(Baseline measure)—completed prior to receiving the first trial cosmetic cover and representing participants’ status when using the cosmesis they had prior to entering the trial.
(2) Outcome measurement 2—completed after 12-weeks of wearing their first test cover, and
(3) Outcome measurement 3—completed after 12-weeks of wearing their second test cover.

Following a telephone discussion with the first author, participants could choose if they wanted to receive an e-mail with a link to a digital survey that could be completed online or if they wished to have the survey sent to their home address in paper form together and with a prepaid return envelope. The online version of the survey was created using Qualtrics software (Qualtrics, Provo, UT). Paper based responses were inputted into Qualtrics, and all data was then exported for analysis.

The first section of the survey was only required to be completed on the first occasion and included information on participants’ sex, age, amputation aetiology, time since amputation and type of cosmetic cover used prior to the study. As personality attributes may affect prosthesis users’ preference for cosmetic cover we also included 8 questions from the BIG-5 Inventory (BFI) which combine to measure participants’ level of extroversion.^23^ Each question in the extroversion dimension of the BFI was measured on a 5-point scale ranging from strongly disagree to agree strongly and, after reversing necessary items (*n* = 3), scores were summed to reflect respondents’ level of extroversion. Higher scores reflecting a high level of extroversion. The Swedish translation of the Big-5 used in this study has been demonstrated to have acceptable levels of reliability and validity for research applications.^24^

Psychosocial well-being of participants was measured using The Amputation Body Image Scale—Refined version (ABIS-R)^25,26^ as well as the psychosocial adjustment subscales from the Trinity Amputation and Prosthesis Experience Scales (TAPES).^27–29^ The ABIS was developed to measure how a person with an amputation perceives their body and scores have been demonstrated to correlate with psychosocial factors such as anxiety, depression, self-esteem and life satisfaction.^25^ The ABIS-R is a Rasch refined version of the original version and comprises of 14 items rated on a 3-point scale (0 = none of the time, 1 = sometimes and 2 = most/all of the time). Two items are reverse scored. A higher score on the ABIS-R reflects a high level of body image disturbance. The ABIS-R has been reported to have good psychometric characteristics.^26^ For the present study the English version was forward translated independently by two Swedish speakers, back translated and reviewed by an expert panel. The TAPES includes three sub-scales related to psychosocial adjustment following a major amputation. These include a general adjustment subscale (5 items), social adjustment subscale (5 items) and a subscale focusing on adjustment to limitations (5 items). Items are rated on a 5-point scale ranging from 1, strongly disagree to 5-strongly agree. Items 9 and 11-15 are reverse scored. Higher scores on each subscale represent greater levels of adjustment. The TAPES and its subscales have been demonstrated to have good reliability and validity.^29^

Participant satisfaction with their device and the services they received were measured using one generic measure, The Quebec User Evaluation of Satisfaction with Assistive Technology 2.0 (QUEST 2.0)^30–32^ as well as measures that were specifically designed for prosthesis users, i.e. satisfaction subscales of the TAPES.^29^ The QUEST 2.0 includes two satisfaction subscales and is designed to be used over a variety of conditions and with a variety of different assistive technologies.^33^ The first subscale comprises of 8 items related to satisfaction with the assistive device while the second comprises of 4 items related to satisfaction with assistive technology services. Items are rated on a 5-point scale from 1, not satisfied at all, to 5, very satisfied. Higher scores represent higher levels of satisfaction. The QUEST 2.0 has good psychometric properties^31,33^ and is available in the Swedish language.^32^

Three subscales of the TAPES address satisfaction with prosthetic devices. Five items address satisfaction with function, four items address satisfaction with aesthetics and 1 item addresses satisfaction with the weight of the device. Each item is measured on a five-point scale (1 = very dissatisfied, 5 = very satisfied) and higher scores are indicative of greater levels of satisfaction. The satisfaction subscales of TAPES have good psychometric properties.^33^ For the present study, we added one item related to hygiene; “How satisfied or dissatisfied are you with the possibility your prosthesis clean”? This item was not addressed in any of the other scales and has been suggested to be superior in 3D printed cosmetic covers.^8^ The item was rated on the same scale as the TAPES items but reported separately.

### Statistical analysis

Descriptive data were calculated using SPSS software. Missing data did not show any obvious pattern and were deemed to be random; no imputation was made. The Shapiro-Wilk’s test and visual inspection of Q–Q plots was used to assess for normality. Data was descriptively analysed on an individual level by comparing the difference in scores that were recorded after testing the foam cover to scores recorded for the same individual after testing the 3D printed cover. Linear mixed effects models (LMM) were then used to assess for changes from the baseline condition including main and interaction effects. Separate analyses were performed for each dependent variable (ABIS-R, QUEST subscales, TAPES subscales and Cleanliness). Subjects were treated as a random effect with a random intercept and the model was fitted using maximum likelihood estimation. Random effects accounted for variability between subjects, and residuals were evaluated to assess model fit. The model analysed the cosmetic cover (i.e. baseline cosmesis, foam cosmesis and 3D printed cosmesis) and the order in which cosmetic covers were tested as fixed effects on the dependent variable. Age, gender, time since amputation and extroversion scores were used as fixed effect covariates. LMM analyses were conducted in R-Studio (version 2022.02.0 build 443) using the lme4 package.^34^ The model specification is as follows: Dependent variable x ∼ Cover * ‘Start cover tested’ + age + gender + years_amp + extrov + (1 | Subject). Confidence intervals for the fixed effects estimates were computed using profile methods. Comparisons between fixed effects were further evaluated using Wald Chi-Squared Analysis of Variance (ANOVA). Post-hoc pairwise comparisons were analyzed with Tukey tests using the “emmeans” package in R.

## Results

**Figure 3.**
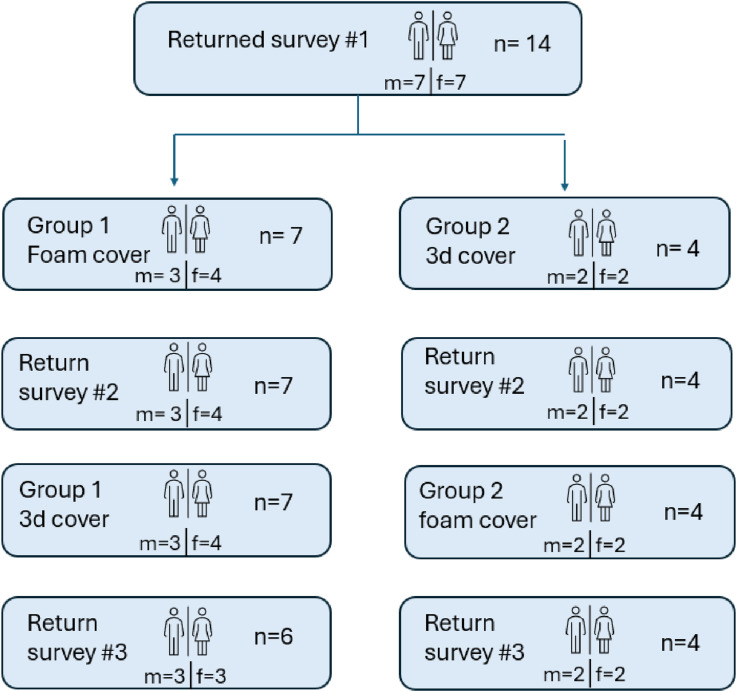
Participant flow chart m = male, f = female.

Data collection took place between August 2023 and August 2024. Fourteen participants were recruited for the study including 7 females and 7 males. Participant details are presented in Table 1. Ages ranged from 30 to 84 (mean 62; SD 16) and the average years since amputation ranged from 2 to 53 years (mean 17; SD 17). 8 participants were amputated at the transtibial level, 2 had undergone a knee disarticulation and 4 were amputated at the transfemoral level. The most common cause of amputation among participants was trauma (*n* = 6). Three had been amputated due to vascular disease, 2 had experienced complications related to an infection, 2 had experienced diabetes related complications and 1 had undergone an amputation because of a tumor. Prior to participating in the study seven participants used a foam cosmetic cover with a nylon or silicon stocking. The remainder used no cover. Figure 3 shows the flow of participants through each stage of the study. Three participants completed the first survey but did not continue with the study after this time. One of these individuals had a change of heart and did not wish to participate while the remaining two were forced to drop-out as the prosthetist who was to provide their device became ill. One participant was unable to be contacted after testing their final cosmetic cover. Outcome measures for this participant were collected for occasions 1 and 2. Outcome measures for all three occasions were collected for all remaining participants (*n* = 10). Upon conclusion of the study 4 participants chose to keep the foam cosmesis that was provided to them, 5 chose to keep the 3D cover and 1 did not wish to use a cosmetic cover at all.

**Table 1.** Participant characteristics.

	Sex	Age	Amputation level	Amputation cause	Time since amputation (years)	Baseline cosmesis - prior to entering study
Group 1	Female	30	KD	Trauma	2,5	No cover
	Male	71	TF	Trauma	51	Foam/ stocking
	Male	45	KD	Tumor	33	No cover
	Male	49	TT	Trauma	6	Foam/ stocking
	Female	47	TT	Diabetes	5	Foam/silicon
	Female	59	TT	Diabetes	7	No cover
	Female**	58	TF	Vascular	2	Foam/ stocking
	Male*	55	TF	Trauma	34	No cover
	Female*	84	TT	Infection	8	Foam/ stocking
Group 2	Female	79	TT	Infection	4	Foam/ stocking
	Male	74	TT	Trauma	19	Foam/ stocking
	Female	76	TT	Vascular	10	No cover
	Male	81	TT	Trauma	6	Foam/silicon
	Male	61	TF	Vascular	53	Foam/ stocking

TT = trans tibial amputation, KD = knee disarticulation amputation, TF = trans femoral amputation.

*Drop-out after completing outcome measures on 1st occasion.

**Did not complete outcome measures on 3rd occasion.

### Descriptive data

Mean results for each outcome measure and cosmetic cover tested are presented as Table 2. When comparing ABIS-R scores after testing the foam cover to scores recorded after testing the 3D printed cover 6 of 10 participants reported better outcomes for the 3D printed cover. One participant reported a better outcome after testing the foam cover and 3 participants recorded identical scores for both covers.

**Table 2.** Results for each outcome measure stratified by the cosmetic cover tested.

	Baseline			Foam			3D		
	Mean	CI lower bound	CI upper bound	Mean	CI lower bound	CI upper bound	Mean	CI lower bound	CI upper bound
ABIS	12.22	6.71	17.74	12.22	7.71	16.73	10.78^#,ɵ^	5.58	15.97
QUEST (device)	29.13	24.14	34.11	27.63	23.05	32.20	29.88	24.11	35.64
QUEST (service)	18.00	16.00	20.00	17.13	14.58	19.67	18.25	16.59	19.91
TAPES (general adjustment)	19.25	14.34	24.16	18.88	14.63	23.12	21.00*^,ɵ^	17.10	24.90
TAPES (social adjustment)	19.88	15.65	24.10	21.38^#^	19.06	23.69	22.43	19.90	24.60
TAPES (adjustment to physical limitations)	12.13	7.62	16.63	12.29^#^	7.27	17.73	11.75	6.54	16.96
TAPES (functional satisfaction)	17.78	15.35	20.20	16.00	13.02	18.98	18.22	14.42	22.02
TAPES (aesthetic satisfaction)	11.22	9.23	13.21	11.44	8.27	14.62	13.78	10.71	16.85
TAPES (weight satisfaction)	2.44	1.58	3.31	2.33	1.18	3.49	2.78	1.71	3.85
Satisfaction with hygiene	3.22	2.38	4.06	2.89	1.91	3.86	3.78	3.27	4.29

* = significant main effect, observed in LMM analysis.

# = significant interaction effect with starting cover, observed in LMM analysis.

¤ = significant main effect, observed in the Wald Chi-Squared ANOVA and confirmed in post hoc analyses.

ɵ = significant interaction effect observed with starting cover, observed in the Wald Chi-Squared ANOVA and confirmed in post hoc analyses.

Device subscale scores on the QUEST resulted in higher (improved) scores after testing the 3D printed cosmesis compared to the foam cosmesis in 6 participants, higher scores after testing the foam cosmesis in 3 participants and an unchanged score for 1 participant. Service subscale scores on the QUEST were higher for the 3D printed cosmesis in 5 participants, higher for the foam cosmesis in 2 participants and unchanged in 1. In this instance data was only available for 8 participants as one participant did not complete all QUEST service subscale questions after testing the foam cosmesis and one participant did not complete all questions after testing the 3D printed cosmesis.

TAPES General Adjustment subscale scores were higher for the 3D printed cosmesis in 5 participants, higher after testing the foam cover in 1 participant and unchanged in 2 participants (note: data was missing for one foam cover survey and one 3D cover survey). Social adjustment scores were higher for the 3D printed cover in 5 participants, higher for the form cover in 3 participants and unchanged in 2. TAPES adjustment to physical limitations scores were higher when testing the foam cover in 6 participants, higher when testing the 3D printed cover in 3 participants and unchanged for one participant.

TAPES functional satisfaction scores were higher after testing the 3D printed cosmesis for 6 participants and higher for the foam cosmesis in 3 cases. One participants failed to complete all necessary questions after testing the 3D printed prosthesis. Satisfaction with aesthetics scores were higher after testing the 3D cover in 7 cases, higher after testing the foam cover in 2 cases and unchanged in 1 case. Satisfaction with weight scores were higher for the 3D cover for 5 participants, higher for the foam cover for 2 participants and unchanged in 3 cases.

When rating the ability to keep the prosthesis clean (hygiene), 6 participants recorded higher scores after testing the 3D cover, 1 recorded a higher score for the foam cover and 4 had the same scores for both conditions.

### LMM and Wald Chi-Squared ANOVA

Tables including both LMM and ANOVA results for each outcome measure are detailed in supplemental data A. Model fits for the fixed effects (Marginal R^2^) varied between 0.20 for TAPES satisfaction with function subscale and 0.33 for hygiene. Model fit when considering both fixed and random effects (Conditional R^2^) ranged between 0.34 for cleanliness to 0.93 for TAPES adjustment to limitations sub scores, suggesting that models explain a moderate to very high proportion of the total variability depending on the outcome variable under investigation.

LMM results for ABIS-R scores showed a significant negative interaction between the 3D cosmetic cover and the order of testing (estimate = −6.38; *p* = .025), i.e. when the UNYQ cover was tested first there was a significant decrease in ABIS-R scores. ANOVA results confirm the interaction effect (χ^2^(2) = 6.95, *p* = .031) with post hoc comparisons indicating a significant difference between the baseline cover and the 3D printed cover (*p* = .04). Other covariates, age, sex and extraversion, were not statistically significant.

No significant main or interaction effects were observed for scores on either of the QUEST sub-scales.

LMM Analysis of scores for the TAPES general adjustment subscale indicated a significant main effect for the 3D cover (estimate = 2.10; *p* = .03) with higher scores recorded for the 3D cosmetic cover compared to baseline values. ANOVA confirmed the main effect (χ^2^(2) = 12.23, *p* = .002) and also recorded an interaction with the order of testing (χ^2^(2) = 6.65, *p* = .04). Post hoc pairwise comparisons revealed that TAPES general scores for the 3D cover were significantly higher than the foam cover when the foam cover was tested first (*p* = .01). Once again the other covariates did not show significant effects.

LMM for TAPES social adjustment subscale revealed a significant interaction effect for cover and order of testing (estimate = 6.060; *p* = .022). This interaction was confirmed by the ANOVA (χ^2^(2) = 7.19, *p* = .03). Post hoc analysis revealed that scores for the foam cover were higher than the baseline measure when the 3D cover was tested first (*p* = .03).

TAPES physical limitations scores also revealed a significant interaction between the foam cover and order of testing (estimate = 5.132; *p* = .008), confirmed by the ANOVA (χ^2^(2) = 9.28, *p* = .009). Post hoc analysis did not identify any significant pairwise differences (*p* > .05).

LMM did not reveal any significant difference for TAPES aesthetics scores however ANOVA results indicated a significant main effect for the 3D cover (χ^2^(2) = 6.39, *p* = .04). Post hoc analysis did not identify any significant difference between the covers (*p* > .05). No significant main or interaction effects were observed or the TAPES function subscale.

ANOVA analysis showed a main effect for cleanliness (χ^2^(2) = 6.35, *p* = .04) however once again post hoc pairwise comparisons did not show significant differences between the covers.

## Discussion

Results of this study provides preliminary evidence to suggest that cosmetic covers representing different design categories (realistic vs expressive) can have a significant effect on the psychosocial well-being of people who are fitted with lower-limb prostheses. On average, scores for psychosocial adjustment (general adjustment) were found to improve when participants were fitted with a 3D printed (expressive) cosmetic cover while body image disturbances decreased. There is some indication that clients’ satisfaction with the aesthetic design and cleanliness of the prosthesis improved with use of the 3D cover. Adjustment to physical limitations appeared to be higher when participants were fitted with the foam (realistic) cosmesis. The covariates sex, age and extroversion scores did not have any significant effect on outcomes tested in this study.

The ABIS-R questionnaire was used in this study to assess how participants perceived and felt about their body. Here it is important to note that lower scores on the ABIS-R reflect more positive results i.e. less body image disturbances. In our study the most promising (i.e. lowest) ABIS-R scores were recorded after testing the 3D printed cover (mean = 10.8). After 12 weeks of testing, ABIS-R scores for those who tested the 3D printed cover first were significantly lower than scores measured at baseline when using the cosmesis they had been prescribed prior to entering the study. The interaction between cosmesis and order of testing if of interest as it may suggest that the context in which a prosthesis is first experienced can influence user perceptions. This may have broader implications for the prescription of cosmetic covers to first time prosthesis users or when switching cover designs in existing users.

ABIS-R scores after testing the 3D printed cover were lower than those reported in a French population of 99 long-term prosthesis users (ABIS-R = 14.6), an Irish sample involving people with diabetes-related amputations (mean = 12.8)^4^ and a Turkish sample of lower limb prosthesis users (mean 12.13).^35^ Unfortunately none of these studies reported on the cosmetic cover design used by participants in their respective studies. In a Turkish sample of people fitted with high-tech prostheses, ABIS-R scores were lower than those reported in the present study (mean 8.1).^12^ This Turkish sample also demonstrated significant differences in the TAPES satisfaction with function subscale when comparing high-tech and lower-tech components, a factor that may well have contributed to the improved ABIS-R scores. In our study, TAPES satisfaction with function scores was not affected by the cover design.

Psychosocial adjustment following amputation is a significant health problem which can affect involvement and performance during rehabilitation and the long term.^13^ In the present study, TAPES sub scores for general psychosocial adjustment differed significantly across designs of cosmetic cover, with scores after testing the 3D cover significantly higher (mean = 21) than the scores after testing the foam cover (mean = 18.9). As was the case with ABIS-R scores, the order of testing influenced results. Scores were comparable to other studies reporting the TAPES general adjustment subscale on samples of lower limb prosthesis users.^4,35,36^ It is important to recognise that the sample of prosthesis users in this study were all two or more years post amputation. Psychosocial health is typically worst in the first two-year post amputation^37^ and results may have had an even greater effect on individuals who were in the early stages of their prosthetic journey. TAPES scores for social adjustment were also affected by cover design and order of testing. While scores were highest for the 3D cover, post hoc tests revealed a significant difference between scores for the foam cover and the baseline measures for participants in group 2.

Average TAPES aesthetic satisfaction scores were highest for the 3D cover (mean = 13.8). ANOVA results indicated a significant overall effect of cosmetic cover design however post hoc tests did not reveal significant differences between pairs of covers. This may be due to the high degree of variability within each group, evident from wide confidence intervals. Given the significant overall findings, we suggest that the relative effects of cosmetic cover design on aesthetic satisfaction should be investigated further with a larger sample size. The highest mean score for aesthetic satisfaction in the present study was considerably lower than scores reported for people with lower-limb diabetes-related amputations in Ireland (mean = 15.4)^4^ and a broader group of lower-limb prostheses from the USA (mean = 15.9).^38^ Both of these studies were conducted over 10 years ago and prior to 3D printed covers becoming widely available. It is difficult to determine why scores are generally lower in the present study but potential reasons could be cultural differences related to the perception of aesthetics, advancements in technology which have led to raised client expectations or changes in design priorities over time.

QUEST has previously been used to assess satisfaction in prosthesis users^39,40^ and has identified significant differences over time in prosthesis users fitted with a microprocessor controlled knee joints.^40^ Our research did not reveal any difference in QUEST scores when comparing the designs of cosmetic cover. The QUEST survey was designed to measure overall user satisfaction and contains a number of items related to dimensions, weight, ease of adjustment and safety and effectiveness as well as items related to service delivery. Our results suggest that aesthetic preferences do not drive satisfaction on these specific items. Functionality with a prosthesis was not evaluated in this study and it is possible that this would have a greater effect on general device satisfaction and satisfaction with services.

Satisfaction with cleanliness has been lifted as an issue with foam covers in the past and recently authors have suggested that 3D printed covers have a superior level of cleanliness.^8^ While our results support this premise, with highest scores recorded for the 3D cosmetic cover, our post hoc comparisons did not reach significance. Further investigation, with a larger sample size, is needed to confirm or refute this result.

3D printed cosmetic are more costly than traditional foam cosmetic covers and possibilities that insurance schemes will cover their costs are reported as worse or much worse than possibilities to receive a foam cosmesis.^8,41^ Unfortunately this makes them less accessible to clients who wish to include them in their prosthetic prescription, creating healthcare equity issues when access becomes restricted to those who can afford out-of-pocket expenses. We argue that potential psychosocial health benefits demonstrated in this study, along with the advantages in durability, ease of attachment, maintenance and reliability highlighted by Efstathiou et al.,^8^ provide evidence for including 3D printed covers in national and private funding schemes for prosthetic users. Selection of a cosmetic cover is however highly personal and 3D printed covers are certainly not the optimal solution for all clients. The high conditional R-squared values in our study indicate that a substantial portion of the variance is due to the random effect of participants, indicating diverse preferences. This aligns with qualitative findings which show that while cosmesis was of paramount importance to some, others may feel ambivalent or even disapproving towards certain types of cosmetic covers.^41^ Prosthetists should subsequently make efforts to educate their clients about the various cosmetic options available to ensure informed decision-making and an optimal solution for the individual.

### Limitations and future research

While our randomised crossover design would have helped to mitigate some concerns related to internal validity (e.g. selection bias, confounding variables), the small number of participants in this study may have impacted on our findings. With only 10 participants completing all three surveys and 1 participant completing 2 of the surveys, we acknowledge that the results may not be generalizable to the broader population of lower limb prosthesis users. We also acknowledge the increased risk of Type II errors, where true effects may go undetected. Future research should aim to address this limitation by including a larger sample of lower limb prosthesis users. A larger scale study would allow for a more robust multivariate analysis, enable researchers to more precisely identify design parameters which affect psychosocial outcomes and to further explore factors that interact to influence outcomes.

Previous work investigating how a silicone cosmesis may affect body image studied a sample of individuals who had already chosen to privately purchase or who had been referred to purchase the cosmetic cover of interest.^13^ This issue was rightly addressed by the authors as a limitation given that participants would have had a pre-existing interest in aesthetics and likely an increased concern about their body image. In this study we attempted to address the issue by excluding individuals who had expressed a desire to receive, or had already chosen to use a 3D cosmetic cover and by recruiting a diverse sample of prosthesis users who would likely have different priorities. As a consequence 9 of the recruited participants had been using a foam cosmetic cover prior to entering the study while 5 had not been using a cover at all. Nevertheless, it is important to acknowledge that participants were aware that the 3D printed cover they received is not typically offered in Sweden and that they received it at no cost. This may have introduced an expectation bias where they saw the 3D cover as being a premium product which should offer significant benefits and may have influenced their response. Knowing that they received something for free that is not typically offered in the Swedish context may also have lead participants to report more favourable outcomes.

While we controlled for a number of covariates and our crossover design would have controlled for static covariates (e.g. socioeconomic status, access to services), other dynamic factors might still have influenced our results. One such factor is the type of cosmetic cover used by participants prior to entering the study which may have biased results. Future work should focus on exploring the impact of this and other unexplored variables, which could include functional status, pain or personal experiences occurring between phases of the study.

While many previous studies report psychosocial wellbeing and satisfaction of prosthesis users, none have reported the cosmetic appearance of devices used by participants. We strongly recommend that future research include information on the aesthetic design of prostheses and consider it as a covariate in analyses. In doing so, researchers can better understand the role of aesthetics in shaping user satisfaction and psychosocial outcomes and provide a more comprehensive view of the factors influencing successful prosthetic care.

Finally we recommend expanding this research with qualitative study designs which can further our understanding of patient preferences and capture a more nuanced perspective on the personal and emotional significance of cosmetic covers.

## Conclusion

Cosmetic covers to alter the aesthetic appearance of a lower limb prosthesis users should not be overlooked as an integral part of the prosthetic prescription process. This research has demonstrated that, while individual preferences vary greatly, cosmetic cover design can have a significant impact on reducing body image disturbances and improving general psychosocial adjustment to the prosthesis. Future research should focus on larger scale studies to better understand the role of aesthetics in shaping user satisfaction and psychosocial outcomes and on better understanding personal preferences of users.

## Supplemental Material

Supplemental Material - Effects of conventional versus 3D-printed cosmetic covers on user satisfaction and psychosocial well-being in lower limb prostheses users: A randomised crossover trial Supplemental Material for Effects of conventional versus 3D-printed cosmetic covers on user satisfaction and psychosocial well-being in lower limb prostheses users: A randomised crossover trial by Nerrolyn Ramstrand, Maria Riveiro, Lars Eriksson and Michael Ceder in Journal of Rehabilitation and Assistive Technologies Engineering

## ORCID iD

Nerrolyn Ramstrand https://orcid.org/0000-0001-8994-8786

## Statements and declarations

## References

[1] Luza LP Ferreira EG Minsky RC , et al. Psychosocial and physical adjustments and prosthesis satisfaction in amputees: a systematic review of observational studies. Disabil Rehabil Assist Technol 2019; 15: 1–8. 10.1080/17483107.2019.1602853 31012753

[2] Ramstrand N Maddock A Johansson M , et al. The lived experience of people who require prostheses or orthoses in the Kingdom of Cambodia: a qualitative study. Disabil Health J 2021; 14: 101071. 33583726 10.1016/j.dhjo.2021.101071

[3] Alessa M Alkhalaf HA Alwabari SS , et al. The psychosocial impact of lower limb amputation on patients and caregivers. Cureus 2022; 14: e31248. 36505108 10.7759/cureus.31248 PMC9731396

[4] Coffey L Gallagher P Horgan O , et al. Psychosocial adjustment to diabetes-related lower limb amputation. Diabet Med 2009; 26: 1063–1067. 19900240 10.1111/j.1464-5491.2009.02802.x

[5] Bekrater-Bodmann R . Factors associated with prosthesis embodiment and its importance for prosthetic satisfaction in lower limb amputees. Front Neurorobot 2021; 14: 604376. 33519413 10.3389/fnbot.2020.604376 PMC7843383

[6] Cairns N Murray K Corney J , et al. Satisfaction with cosmesis and priorities for cosmesis design reported by lower limb amputees in the United Kingdom: instrument development and results. Prosthet Orthot Int 2014; 38: 467–473. 24327666 10.1177/0309364613512149 PMC4230545

[7] Xian O Irawan I . A case study on identifying the aesthetic values of lower limb prosthetic. In Proceedings of the International conference on design industries and creative culture. Design Decoded, 2019.

[8] Efstathiou K McGarry A . 3D printed cosmetic covers for lower limb prosthetics. Can Prosthet Orthot J 2023; 6: 42176. 38873131 10.33137/cpoj.v6i2.42176 PMC11168597

[9] Ramstrand N Riveiro M Eriksson L , et al. Designing feelings into lower-limb prostheses—a kansei engineering approach to understand lower-limb prosthetic cosmeses. J Rehabil Assist Technol Eng 2024; 11: 20556683241289938. 39430059 10.1177/20556683241289938 PMC11489910

[10] Amplitude Media Group . The most affordable 3D-printed prosthesis covers for amputees. Amplitude, 2022.

[11] Murray CD Fox J . Body image and prosthesis satisfaction in the lower limb amputee. Disabil Rehabil 2002; 24: 925–931. 12519488 10.1080/09638280210150014

[12] Burçak B Kesikburun B Köseoğlu BF , et al. Quality of life, body image, and mobility in lower-limb amputees using high-tech prostheses: a pragmatic trial. Ann Phys Rehabil Med 2021; 64: 101405. 32561506 10.1016/j.rehab.2020.03.016

[13] Donovan-Hall MK Yardley L Watts RJ . Engagement in activities revealing the body and psychosocial adjustment in adults with a trans-tibial prosthesis. Prosthet Orthot Int 2002; 26: 15–22. 12043922 10.1080/03093640208726617

[14] Atherton R Robertson N . Psychological adjustment to lower limb amputation amongst prosthesis users. Disabil Rehabil 2006; 28: 1201–1209. 17005481 10.1080/09638280600551674

[15] Highsmith MJ Kahle JT Knight M , et al. Delivery of cosmetic covers to persons with transtibial and transfemoral amputations in an outpatient prosthetic practice. Prosthet Orthot Int 2016; 40: 343–349. 25575552 10.1177/0309364614564024

[16] Randolph MG Elbaum L Wen PS , et al. Functional and psychosocial status of Haitians who became users of lower extremity prostheses as a result of the 2010 earthquake. J Prosthet Orthot 2014; 26: 177–182. 25554722 10.1097/jpo.0000000000000039 PMC4278370

[17] Vlachaki A Paterson AMJ Porter SC , et al. Exploring users’ attitudes towards prosthesis aesthetics in the UK and Greece. Design for Health 2020; 4: 4–23.

[18] Hall ML Orzada BT . Expressive prostheses: meaning and significance. Fash Pract 2013; 5: 9–32.

[19] UNYQ guides. BK - skanning. https://unyq.com/measurement-guides (accessed 3 October 2024).

[20] Anatomic Studios . How it works. https://anatomic-studios.com/how-it-works/ (accessed 3 October 2024).

[21] Jones B Kenward MG . Design and analysis of cross-over trials. Chapman and Hall/CRC, Monographs On Statistics And Applied Probability, 2003.

[22] Lim CY In JJ . Considerations for crossover design in clinical study. Korean J Anesthesiol 2021; 74: 293–299. 34344139 10.4097/kja.21165 PMC8342834

[23] John OP Srivastava S . The Big-Five trait taxonomy: history, measurement, and theoretical perspectives. In: Pervin LA John OP (eds). Handbook of personality: Theory and Research. 2nd ed. Guilford Press, 1999, pp. 102–138.

[24] Zakrisson I . Big five inventory (BFI): utprövning för svenska förhållanden. Mid Sweden university. Östersund, 2010.

[25] Breakey JW . Body image: the lower-limb amputee. JPO J Prosthetics Orthot 1997; 9: 58–66.

[26] Gallagher P Horgan O Franchignoni F , et al. Body image in people with lower-limb amputation: a rasch analysis of the amputee body image scale. Am J Phys Med Rehabil 2007; 86: 205–215. 17314705 10.1097/PHM.0b013e3180321439

[27] Gallagher P Franchignoni F Giordano A , et al. Trinity amputation and prosthesis experience scales: a psychometric assessment using classical test theory and rasch analysis. Am J Phys Med Rehabil 2010; 89: 487–496. 20489393 10.1097/PHM.0b013e3181dd8cf1

[28] Gallagher P Maclachlan M . The Trinity Amputation and Prosthesis Experience Scales and quality of life in people with lower-limb amputation. Arch Phys Med Rehabil 2004; 85: 730–736. 15129396 10.1016/j.apmr.2003.07.009

[29] Gallagher P MacLachlan M . Development and psychometric evaluation of the trinity amputation and prosthesis experience scales (TAPES). Rehabil Psychol 2000; 45: 130–154.

[30] Demers L Weiss-Lambrou R Ska B . Development of the Quebec user evaluation of satisfaction with assistive technology (QUEST). Assist Technol 1996; 8: 3–13. 10159726 10.1080/10400435.1996.10132268

[31] Demers L Weiss-lambrou R Ska B . The Quebec user evaluation of satisfaction with assistive technology (QUEST 2.0): an overview and recent progress. Technol Disabil 2002; 14: 101–105.

[32] Wressle E Samuelsson K . Testing the Swedish version of the Quebec user evaluation of satisfaction with assistive technology (QUEST) 2.0. Assist Technol: The Official Journal of RESNA. 2003; 927–930.

[33] Demers L Weiss-Lambrou R Ska B . Item analysis of the Quebec user evaluation of satisfaction with assistive technology (QUEST). Assist Technol 2000; 12: 96–105. 11508406 10.1080/10400435.2000.10132015

[34] Bates D Mächler M Bolker B , et al. Fitting linear mixed-effects models using lme4. J Stat Software 2015; 67: 1–48.

[35] Desteli EE İmren Y Erdoğan M , et al. Comparison of upper limb amputees and lower limb amputees: a psychosocial perspective. Eur J Trauma Emerg Surg 2014; 40: 735–739. 26814792 10.1007/s00068-014-0418-3

[36] Desmond D Gallagher P Henderson-Slater D , et al. Pain and psychosocial adjustment to lower limb amputation amongst prosthesis users. Prosthet Orthot Int 2008; 32: 244–252. 18569892 10.1080/03093640802067046

[37] Horgan O MacLachlan M . Psychosocial adjustment to lower-limb amputation: a review. Disabil Rehabil 2004; 26: 837–850. 15497913 10.1080/09638280410001708869

[38] Webster JB Hakimi KN Williams RM , et al. Prosthetic fitting, use, and satisfaction following lower-limb amputation: a prospective study. J Rehabil Res Dev 2012; 49: 1493–1504. 23516053 10.1682/jrrd.2012.01.0001

[39] Kablan N Bakhsh HR Alammar W , et al. Psychometric evaluation of the Arabic version of the Quebec user evaluation of satisfaction with assistive technology (A-QUEST 2.0) in prosthesis users. Eur J Phys Rehabil Med 2022; 58: 118–126. 34247472 10.23736/S1973-9087.21.06880-5 PMC9980568

[40] Lansade C Chiesa G Paysant J , et al. Impact of C-LEG on mobility, satisfaction and quality of life in a multicenter cohort of femoral amputees. Ann Phys Rehabil Med 2021; 64: 101386. 32360291 10.1016/j.rehab.2020.03.011

[41] Murray CD . Being like everybody else: the personal meanings of being a prosthesis user. Disabil Rehabil 2009; 31: 573–581. 19034778 10.1080/09638280802240290

