# Supplementary material for: Effects of conventional versus 3D-printed cosmetic covers on user satisfaction and psychosocial well-being in lower limb prostheses users: A randomised crossover trial
Source: J Rehabil Assist Technol Eng. 2025 Apr 4;12:20556683251330996. doi: 10.1177/20556683251330996 (PMC12032455; doi:10.1177/20556683251330996)
Supplement: Supplemental Material - Effects of conventional versus 3D-printed cosmetic covers on user satisfaction and psychosocial well-being in lower limb prostheses users: A randomised crossover trial [file sj-pdf-1-jrt-10.1177_20556683251330996.pdf]

<u>Supplementary data A</u>

LMM results for ABIS

**Fixed effects**								
	Est/Beta	SE	t	CI lower	CI upper	P value	Chi-Square	Pr(>Chisq)
(Intercept)	1,340	17,923	0,075	-25,466	28,291	0,943	0.006	0.940
Cover (foam)	1,592	1,377	1,156	-1,045	4,019	0,266		
Cover (3D)	0,696	1,459	0,477	-1,958	3,385	0,640	1.342	0.511
Start cover	5,355	6,543	-0,616	-11.265	4.752	0,559	0.380	0.538
Age	0,247	0,180	1,376	-0,022	0,517	0,217	1.893	0.169
Gender	2,121	5,553	0,382	-6,181	10,453	0,716	0.146	0.702
Years with amp	-0,146	0,159	-0,918	-0,384	0,092	0,393	0.843	0.358
Extraversion	-0,072	0,490	-0,146	-0,809	0,660	0,889	0.021	0.884
Cover:Start cover (foam)	-4,947	2,514	-1,968	-9,476	-0,261	0,068		
Cover;start cover (3d)	-6,384	2,562	-2,492	-11,125	-1.731	0,025*	6.954	0.031*

**Random effects**		
	Variance	SD
Participant (Intercept)	34.21	5.85
Residual variance	6.69	2.58

**Model fit**	
Marginal R^2	Conditional R^2

0.24	0.88

QUEST satisfaction with device

Fixed effects								
	Estimate	Std. Error	t value	95% CI Lower	95% CI Upper	p-value	Chi-Square	Pr(>Chisq)
(Intercept)	24.79233	16.12080	1.538	0.5111	49.1922	0.171	2.3652	0.1241
Cover (foam)	-5.39257	5.68686	-0.948	-7.0146	1.4019	0.358		
Cover (3d)	-5.39636	5.91283	-0.913	-5.908	3.2416	0.376	1.1773	0.5062
Start cover	-0.13105	4.51159	-0.029	-6.9768	6.9505	0.977	0.0008	0.9768
Age	0.10632	0.13435	0.791	-0.1029	0.3077	0.453	0.6262	0.4287
Gender	-3.41914	5.07480	-0.674	-11.1544	4.1572	0.527	0.4539	0.5005
Years amp	0.04215	0.14922	0.282	-0.1864	0.2655	0.786	0.0798	0.7776
Extraversion	-0.01642	0.45527	-0.036	-0.6984	0.6740	0.972	0.0013	0.9712
Cover:Start cover (foam)	2.82389	4.12757	0.684	-5.1387	10.2815	0.504		
Cover:Start cover (3d)	4.65912	4.21032	1.107	-3.1207	12.6339	0.286	1.2546	0.5340

Random effects		
	Variance	SD
Participant (Intercept)	23.99	4.898
Residual variance	18.32	4.280

Model fit	
Marginal R^2	Conditional R^2

0.242	0.672

QUEST satisfaction with service

Fixed effects								
	Est/Beta	SE	t	CI lower	CI upper	P value	Chi-Square	Pr(>Chisq)
(Intercept)	6.723	5.736	1.172	-1.857	15.377	0.282	1,3736	0,2412
Cover (foam)	0.469	1.669	0.402	-1.4884	2.7555	0.693		
Cover (3d)	0.322	1.171	0.275	-1.9443	2.312	0.787	0,1749	0,9163
Start cover	1.75838	1.75645	1.001	-0.9520	4.5425	0.336	1.0022	0.3168
Age	0.01389	0.05000	0.278	-0.0638	0.0888	0.788	0.0771	0.7812
Gender	0.96806	1.79529	0.539	-1.7244	3.6449	0.610	0.2908	0.5897
Years amp	0.05231	0.05395	0.969	-0.0285	0.1338	0.365	0.9398	0.3323
Extraversion	0.30767	0.16308	1.887	0.0637	0.5524	0.106	3.5592	0.0592
Cover:Start cover (foam)	-2.51601	2.07188	-1.214	-6.4180	1.1089	0.243		
Cover:Start cover (3d)	0.29178	2.28993	0.127	-3.9276	4.2788	0.900	1.9653	0.3743
**Random effects**								
	Variance			SD				
Participant (Intercept)	2.124			1.457				

Residual variance	4.537	2.130
**Model fit**		
Marginal R^2		Conditional R^2
0.255		0.493

TAPES psychological adjustment - General adjustment

Fixed effects

	Est/Beta	SE	t	CI lower	CI upper	P value	Chi-Square	Pr(>Chisq)
(Intercept)	8,570	12,239	0,700	-10.426	27.560	0,506	0.490	0.484
Cover (foam)	-0,857	0,810	-1,059	-2.327	0.613	0,307		
Cover (3d)	2,103	0,859	2,450	-0.503	3.625	0,028*	12.231	0.002**
Start cover	-1,637	3,219	-0,508	-6.640	3.371	0,625	0.258	0.611
Age	0,019	0,100	0,193	-0.136	0.174	0,852	0.037	0.847
Gender	-1,480	3,990	-0,371	-7.676	4.703	0,722	0.138	0.711
Years amp	0,113	0,114	0,993	-0.064	0.290	0,353	0.986	0.321
Extraversion	0,367	0,353	1,039	-0.181	0.915	0,333	1.080	0.299
Cover:Start cover (foam)	2,793	1,480	1,887	0.057	5.440	0,080		
Cover:Start cover (3d)	-1,274	1,685	-0,756	-4.426	1.704	0,462	6.651	0.036*

Random effects

	Variance	SD
Participant (Intercept)	18.320	4.280
Residual variance	2.312	1.521

Model fit

Marginal R^2	Conditional R^2
0.299	0.921

TAPES psychological adjustment - Social adjustment

Fixed effects								
	Est/Beta	SE	t	CI lower	CI upper	P value	Chi-Square	Pr(>Chisq)
(Intercept)	11.272	9.462	1.191	-3.049	25.560	0.276	1.419	0.234
Cover (foam)	-0.196	1.294	-0.151	-2.642	2.167	0.882	2.002	0.368
Cover (3d)	1.619	1.371	1.181	-1.190	3.974	0.255		
Start cover	-1.955	2.663	-0.734	-6.016	2.191	0.481	0.539	0.463
Age	-0.003	0.080	-0.035	-0.125	0.117	0.973	0.001	0.972
Gender	0.764	3.018	0.253	-3.855	5.281	0.809	0.064	0.800
Years amp	0.061	0.089	0.689	-0.075	0.194	0.515	0.474	0.491
Extraversion	0.328	0.271	1.213	-0.077	0.741	0.270	1.471	0.225
Cover:Start cover (foam)	6.060	2.373	2.554	1.314	10.211	0.022*	7.187	0.028*
Cover:Start cover (3d)	1.245	2.420	0.514	-3.384	5.648	0.614		
**Random effects**								
	Variance	SD						
Participant (Intercept)	8.650	2.941						

Residual variance	6.047	2.459
**Model fit**		
Marginal R^2		Conditional R^2
0.210		0.675

TAPES psychological adjustment – adjustment to limitations

**Fixed effects**								
	Est/Beta	SE	t	CI lower	CI upper	P value	Chi-Square	Pr(>Chisq)
(Intercept)	38.601	14.751	2.617	15.677	61.526	0.034	6.848	0.009*
Cover (foam)	-1.866	0.921	-2.024	-3.553	-0.192	0.061		
Cover (3d)	-1.484	0.978	-1.519	-3.311	0.258	0.149	4.513	0.104
Start cover	2.411	3.872	0.623	-3.615	8.441	0.551	0.388	0.533
Age	-0.166	0.120	-1.379	-0.353	0.021	0.208	1.902	0.168
Gender	-1.742	4.811	-0.362	-9.221	5.725	0.728	0.131	0.717
Years amp	0.103	0.137	0.748	-0.111	0.316	0.478	0.560	0.454
Extraversion	-0.654	0.426	-1.536	-1.315	0.008*	0.168	2.360	0.124
Cover:Start cover (foam)	5.132	1.685	3.045	2.016	8.167	0.008*		
Cover:Start cover (3d)	2.418	1.717	1.408	-0.720	5.546	0.179	9.284	0.010*
**Random effects**								

	Variance	SD
Participant (Intercept)	26.800	5.177
Residual variance	2.996	1.731
**Model fit**		
Marginal R^2		Conditional R^2
0.306		0.930

TAPES Satisfaction with prosthesis - aesthetics

**Fixed effects**								
	Est/Beta	SE	t	CI lower	CI upper	P value	Chi-Square	Pr(>Chisq)
(Intercept)	10.322	8.1444	1.267	-1.713	22.286	0.2524	1.606	0.205
Cover (foam)	-1.783	1.615	-1.104	-4.823	1.174	0.285		
Cover (3d)	2.630	1.708	1.540	-0.8125	5.518	0.142	6.389	0.041*
Start cover	-1.198	2.517	-0.476	-5.035	2.865	0.642	0.226	0.634
Age	0.013	0.071	0.187	-0.096	0.118	0.856	0.035	0.852
Gender	-2.333	2.531	-0.922	-6.171	1.319	0.399	0.850	0.357
Years amp	0.015	0.077	0.199	-0.102	0.127	0.848	0.040	0.842
Extraversion	0.075	0.230	0.328	-0.257	0.422	0.755	0.108	0.743
Cover:Start cover (foam)	5.326	2.972	1.792	-0.510	10.545	0.092	4.198	0.123

Cover:Start cover (3d)	-0.088	3.035	-0.029	-5.799	5.498	0.977		
**Random effects**								
	Variance			SD				
Participant (Intercept)	4.065			2.016				
Residual variance	9.691			3.113				
**Model fit**								
Marginal R^2				Conditional R^2				
0.257				0.476				

TAPES Satisfaction with prosthesis – weight

Fixed effects								
	Est/Beta	SE	t	CI lower	CI upper	P value	Chi-Square	Pr(>Chisq)
(Intercept)	6.115	3.100	1.973	1.792	10.464	0.105	3.892	0.0485*
Cover (foam)	-0.213	0.486	-0.437	-1.264	0.693	0.668		
Cover (3d)	0.046	0.515	-0.090	-1.172	0.904	0.929	0.408	0.862
Start cover	0.106	0.897	0.118	-1.179	1.471	0.909	0.014	0.906
Age	0.013	0.026	0.505	-0.025	0.050	0.631	0.255	0.614
Gender	-1.857	0.981	-1.894	-3.298	-0.525	0.124	3.587	0.058
Years amp	-0.025	0.029	-0.873	-0.071	0.014	0.421	0.762	0.383
Extraversion	-0.102	0.088	-1.160	-0.223	0.024	0.301	1.345	0.246
Cover:Start cover (foam)	0.562	0.893	0.629	-1.546	2.070	0.539		
Cover:Start cover (3d)	0.303	0.911	0.332	-1.728	1.957	0.745	0.397	0.820

Random effects		
	Variance	SD
Participant (Intercept)	0.829	0.911
Residual variance	0.861	0.928

Model fit	
Marginal R^2	Conditional R^2
0.313	0.650

TAPES Satisfaction with prosthesis – function

Fixed effects								
	Est/Beta	SE	t	CI lower	CI upper	P value	Chi-Square	Pr(>Chisq)
(Intercept)	11.148	11.848	0.941	-6.754	28.913	0.382	0.885	0.347
Cover (foam)	-2.944	1.514	-1.944	-5.734	-0.075	0.072		
Cover (3d)	0.106	1.722	0.062	--3.054	3.361	0.951	4.760	0.092
Start cover	-1.275	3.299	-0.386	-6.265	3.820	0.709	0.149	0.699
Age	0.107	0.099	1.083	-0.045	0.256	0.314	1.173	0.279
Gender	-1.299	3.797	-0.342	-7.052	4.370	0.745	0.117	0.732
Years amp	0.012	0.111	0.110	-0.155	0.178	0.916	0.012	0.912
Extraversion	0.027	0.340	0.080	-0.480	0.546	0.939	0.007	0.936
Cover:Start cover (foam)	4.335	2.776	1.561	-1.105	9.322	0.141		
Cover:Start cover (3d)	-0.049	2.902	-0.017	-5.740	5.172	0.987	3.168	0.205

Random effects		
	Variance	SD
Participant (Intercept)	14.136	3.760
Residual variance	8.253	2.873

Model fit	
Marginal R^2	Conditional R^2
0.1992	0.705

Satisfaction with ability to keep clean

Fixed effects								
	Est/Beta	SE	t	CI lower	CI upper	P value	Chi-Square	Pr(>Chisq)
(Intercept)	4.115	1.436	2.867	1.777	6.482	0.027	0.218	0.004*
Cover (foam)	-0.800	0.387	-2.066	-1.456	-0.150	0.054		
Cover (3d)	0.162	0.408	0.398	-0.532	0.843	0.695	6.345	0.042*
Start cover	0.664	0.507	1.310	-0.174	1.514	0.207	1.716	0.190
Age	-0.017	0.013	-1.317	-0.039	0.004	0.221	1.734	0.188
Gender	0.093	0.432	0.216	-0.615	0.794	0.838	0.047	0.829
Years amp	-0.008	0.014	-0.583	-0.030	0.014	0.579	0.340	0.560
Extraversion	0.013	0.040	0.335	-0.051	0.078	0.750	0.112	0.738
Cover:Start cover (foam)	1.014	0.714	1.420	-0.189	2.221	0.173		
Cover:Start cover (3d)	0.051	0.729	0.070	-1.174	1.288	0.945	2.449	0.294

Random effects		
	Variance	SD
Participant (Intercept)	0.015	0.120
Residual variance	0.575	0.758

Model fit	
Marginal R^2	Conditional R^2
0.332	0.349
